# Association of elastic power in mechanical ventilation with the severity of acute respiratory distress syndrome: a retrospective study

**DOI:** 10.1186/s40001-023-01577-7

**Published:** 2024-01-03

**Authors:** Yongpeng Xie, Jiaxin Shi, Suxia Liu, Xiaobing Chen, Yanli Wang, Xiaomin Li, Yao Yan

**Affiliations:** 1 Department of Emergency and Critical Care Medicine, Lianyungang Clinical College of Nanjing Medical University, Lianyungang, 222000 Jiangsu China; 2 Department of Respiratory and Critical Care Medicine, Lianyungang Clinical College of Nanjing Medical University, Lianyungang, 222000 Jiangsu China; 3 Department of Critical Care Medicine, The Second people,s Hospital of Lianyungang City, Lianyungang, 222000 Jiangsu China

**Keywords:** Acute respiratory distress syndrome, Mechanical ventilation, Elastic power, Mechanical power, Severity assessment

## Abstract

**Background:**

Mechanical power (MP) is the total energy released into the entire respiratory system per minute which mainly comprises three components: elastic static power, Elastic dynamic power and resistive power. However, the energy to overcome resistance to the gas flow is not the key factor in causing lung injury, but the elastic power (EP) which generates the baseline stretch of the lung fibers and overcomes respiratory system elastance may be closely related to the ARDS severity. Thus, this study aimed to investigate whether EP is superior to other ventilator variables for predicting the severity of lung injury in ARDS patients.

**Methods:**

We retrieved patient data from the Medical Information Mart for Intensive Care III (MIMIC-III) database. The retrieved data involved adults (≥ 18 years) diagnosed with ARDS and subjected to invasive mechanical ventilation for ≥ 48 h. We employed univariate and multivariate logistic regression analyses to investigate the correlation between EP and development of moderate-severe ARDS. Furthermore, we utilized restricted cubic spline models to assess whether there is a linear association between EP and incidence of moderate-severe ARDS. In addition, we employed a stratified linear regression model and likelihood ratio test in subgroups to identify potential modifications and interactions.

**Results:**

Moderate-severe ARDS occurred in 73.4% (296/403) of the patients analyzed. EP and MP were significantly associated with moderate-severe ARDS (odds ratio [OR] 1.21, 95% confidence interval [CI] 1.15–1.28, *p* < 0.001; and OR 1.15, 95%CI 1.11–1.20, *p* < 0.001; respectively), but EP showed a higher area-under-curve (95%CI 0.72–0.82, *p* < 0.001) than plateau pressure, driving pressure, and static lung compliance in predicting ARDS severity. The optimal cutoff value for EP was 14.6 J/min with a sensitivity of 75% and specificity of 66%. Quartile analysis revealed that the relationship between EP and ARDS severity remained robust and reliable in subgroup analysis.

**Conclusion:**

EP is a good ventilator variable associated with ARDS severity and can be used for grading ARDS severity. Close monitoring of EP is advised in patients undergoing mechanical ventilation. Additional experimental trials are needed to investigate whether adjusting ventilator variables according to EP can yield significant improvements in clinical outcomes.

**Supplementary Information:**

The online version contains supplementary material available at 10.1186/s40001-023-01577-7.

## Introduction

Acute respiratory distress syndrome (ARDS), as defined by the Berlin criteria, is a complex syndrome characterized by acute hypoxic respiratory failure resulting from various insults [1]. Mechanical ventilation is recommended for ARDS patients to avoid life-threatening hypoxia and hypercapnia; however, it is associated with ventilator-induced lung injury (VILI) [2]. Currently, there is a lack of respiratory mechanics monitoring and evaluation indicators for the severity of lung injury in the Berlin criteria for ARDS [3]. Accurately grading ARDS severity and providing precise lung-protective ventilation therapy are essential for preventing VILI and improving survival in ARDS [4]. Mechanical power (MP), a comprehensive energy index based on various ventilator parameters, that can be included in any ventilator to monitor the safety of mechanical ventilation and guide lung protective strategies [5]. MP is the total energy released into the entire respiratory system per minute which mainly obtained as the algebraic sum of three components: elastic static power related to PEEP, Elastic dynamic power related to driving pressure and resistive power related to resistance in the ventilator circuit, endotracheal tube, and airways [6]. Empirical evidence indicates that MP is associated with mortality in mechanically ventilated patients [7, 8]. However, the energy to overcome resistance to the gas flow is not the key factor in causing lung injury, but the elastic power (EP) which generates the baseline stretch of the lung fibers and overcomes respiratory system elastance may be closely related to the ARDS severity. Therefore, we hypothesized that EP is more sensitive to "catch" the worsening respiratory mechanics as compared to other variables. Thus, this study aimed to assess the discriminatory power of EP in predicting the severity of lung injury in ARDS patients.

## Methods

We conducted a observational retrospective study [9] using electronic health-records data from the Medical Information Mart for Intensive Care-III v1.4 (MIMIC-III v1.4) [10]. The MIMIC-III v1.4 database contains comprehensive and high-quality data of well-defined and characterized patients admitted to the intensive care unit (ICU) at the Beth Israel Deaconess Medical Center between 2001 and 2012. One author (YY) accessed the database and extracted the data (certification number 41699414). All the analyses were carried out in accordance with the relevant guidelines and regulations. The data in MIMIC-III were de-identified, and the use of the database for research was approved by the Institutional Review Boards of the Massachusetts Institute of Technology and Beth Israel Deaconess Medical Center.

### Patient selection

For this study, 61,532 ICU admissions were screened for patients who received invasive mechanical ventilation for ≥ 48 h. The inclusion criteria complied with the 2012 ARDS Berlin diagnostic criteria. The exclusion criteria were as follows: Patients (1) not being admitted to the hospital and ICU for the first time, (2) aged < 18 years, (3) who were extubated or died within 48 h of admission to the ICU, (4) who received extracorporeal membrane oxygenation, (5) whose PaO_2_/FiO_2_ ratio was unavailable, or (6) whose data on ventilation variables required for MP calculation were missing were excluded (i.e. only volume-controlled ventilation patients were included, not pressure-controlled or supported modes). According to the lowest PaO_2_/FiO_2_ ratio in the first 24 h of ventilation. the patients were divided into mild-moderate (> 150 mmHg) and moderate-severe (≤ 150 mmHg) ARDS groups.

### Data collection

Data were extracted from the database by using Structured Query Language in pgAdmin 4.3. We extracted the following variables: (1) basic demographics, including age, gender, weight, and height; (2) disease severity, which was defined at ICU admission by using the sequential organ failure assessment (SOFA) score or simplified acute physiology score II (SAPS II); (3) ARDS etiology and comorbidities; (4) intervention measures within 24 h of ICU admission, including vasoactive drug administration and renal replacement therapy; (5) physiological variables in the first 24 h of ventilation; (6) respiratory mechanics parameters within 48 h of ventilation, including tidal volume (VT), positive-end expiratory pressure (PEEP), plateau pressure (Pplat), peak inspiratory pressure (Ppeak), driving pressure (ΔP), respiratory rate (RR), static lung compliance (Cst), and inspired oxygen fraction (FiO_2_); and (7) clinical outcomes, including duration of invasive mechanical ventilation (IMV), ICU length of stay (LOS), and hospital LOS. All ventilation variables were extracted as the highest, lowest and average values of each 6-h time frame during the 48 h of ventilation, and the average of the highest and lowest 6-h time frame mean values was calculated to obtain the 24-h ventilation variables.

**Calculation of EP**

We obtained the derived variables according to the simplified MP equation in the volume-controlled ventilation mode proposed by Gattinoni. The equation used for the calculation is as follows:

MP (J/min) = 0.098 × VT × RR × (Ppeak – ½ΔP).

The ∆P in the ventilation mode was calculated using Pplat and PEEP as follows:

∆P (cmH_2_O) = Pplat ‒ PEEP.

The Cst was calculated using VT and ΔP as follows:

Cst = VT / ΔP.

EP was calculated as follows (Additional file 1: Fig. S1):

EP (J/min) = 0.098 × VT × RR × ½ (PEEP + Pplat).

The 24-h EP is the average of the highest average EP and the lowest average EP. Given that the parameters gradually stabilized after 24 h of ventilation, we used the parameters from the second 24 h of ventilation for data analysis. [11]

### Definitions and outcomes

ARDS severity was defined according to the Berlin definition, with the PaO_2_/FiO_2_ thresholds > 150 mmHg for mild-moderate ARDS and ≤ 150 mmHg for moderate-severe ARDS. In the MIMIC-III database, ARDS was assigned based on the following criteria: (1) acute onset within 1 week; (2) chest radiograph showing bilateral lung opacities or infiltration in noteevents; (3) PO_2_/FiO_2_ ratio ≤ 300 mmHg; and (4) PEEP ≥ 5 cmH_2_O to exclude respiratory failure caused by cardiogenic factors or fluid overload. The outcome event was the development of moderate-severe ARDS among those with ARDS.

### Statistical analysis

Patient characteristics were calculated according to EP quartiles. Continuous variables with normal or skewed distributions were described as mean ± standard deviation (SD) and interquartile range (IQR). Categorical variables were expressed as numbers (percentages). Comparisons between groups were made using the Student’s t-test or Kruskal–Wallis test for continuous variables and the chi-square test or Fisher’s exact test for categorical variables as appropriate. Missing data were imputed via multiple imputation (detailed in supplementary) [12].

The association between EP and moderate-severe ARDS was investigated using univariate and multivariate logistic regression analyses. Variables with p < 0.1 at univariate analysis and variables without co-linearity were included as covariates for multivariate analysis. We applied three models in the regression analysis. The multivariable models were adjusted as follows: model 1 was adjusted for age and body mass index (BMI); model 2 was adjusted for model 1 plus SOFA score; and model 3 was adjusted for model 2 plus MBP and PCO_2_.

We used restricted cubic spline models to examine the possible linear association between EP and the incidence of moderate-severe ARDS [13]. The analysis took into account the covariates mentioned above (model 3), and the median value of EP was set as the reference point. A knot was placed at the 5th, 35th, 65th, and 95th percentiles of EP.

To identify modifications and interactions, we used a stratified linear regression model and likelihood ratio test in subgroups of age (< 65 or ≥ 65 years), gender (female or male), BMI (< 28 or ≥ 28 kg/m^2^), SOFA score (< 7 or ≥ 7), respiratory disease (yes or no), and sepsis (yes or no). Additional subgroup analyses were performed when EP was treated as a categorical variable in separate models. In the sensitivity analyses, a complete case analysis was conducted under the assumption that data were missing (Additional file 1: Table S1) completely at random. Given the potential effect of time of illness on observed associations, we conducted an additional sensitivity analysis by using the data from the first 24 h of ventilation. A two-tailed test was performed, and p < 0.05 was considered to indicate statistical significance. All the analyses were performed using the statistical software R (version 4.1.2; http://www.R-project.org; The R Foundation) and Free Statistics (version 1.7).

## Results

### Baseline characteristics of the study population

**Fig. 1:**
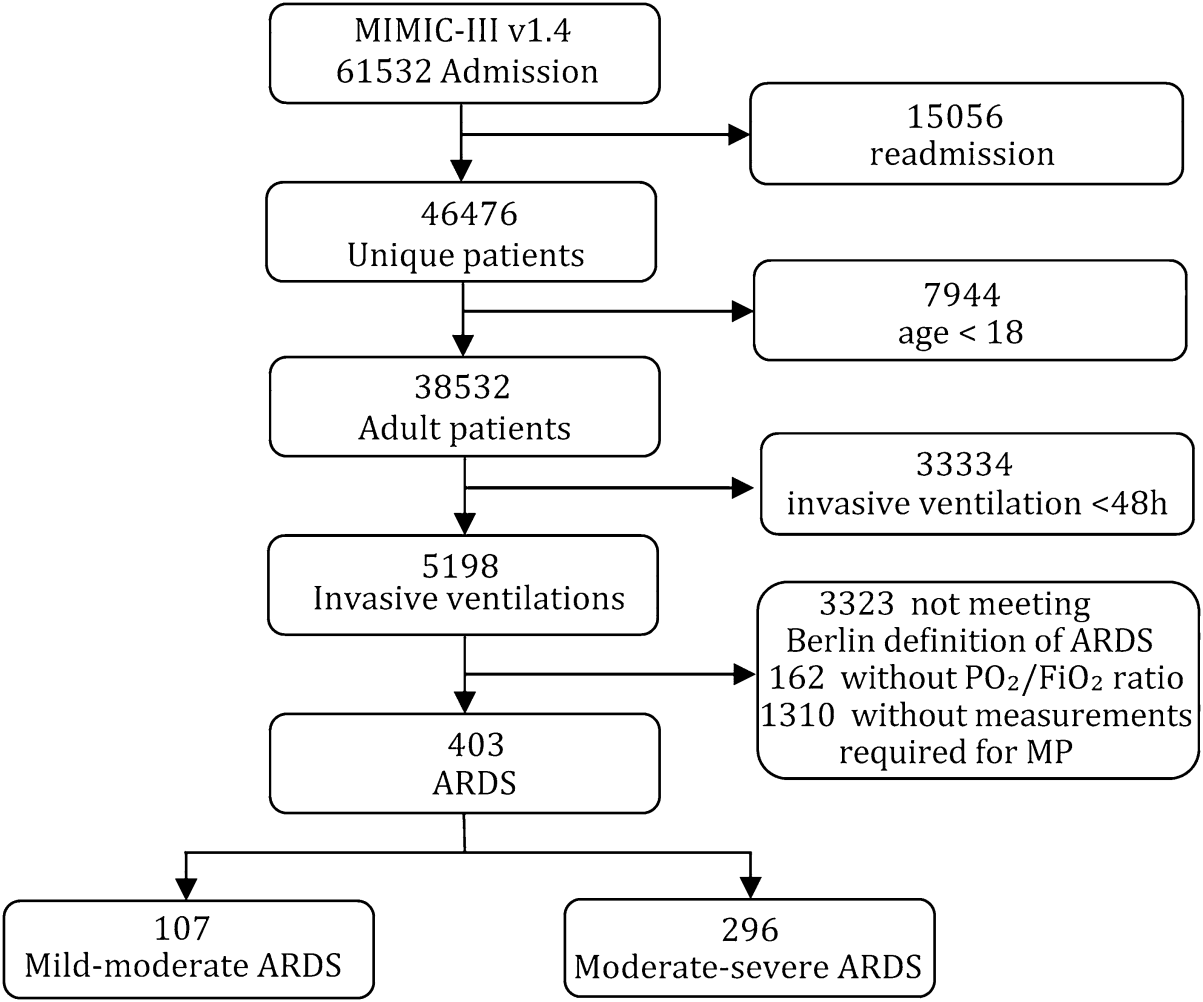
Flow chart of the study population

**Fig. 2:**
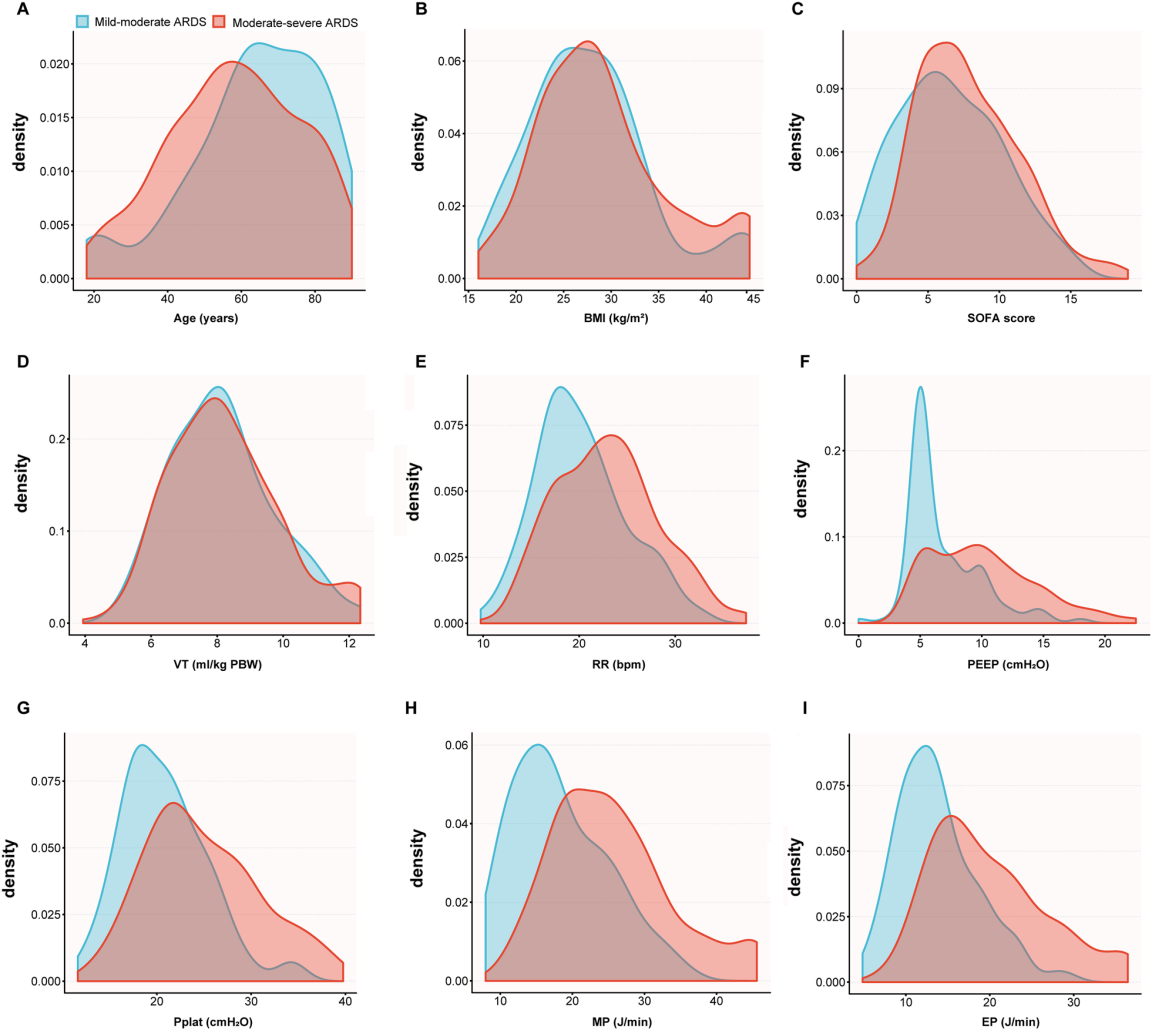
The distribution of baseline characteristics and ventilatory variables between mild-moderate ARDS and moderate-severe ARDS. The area shaded with blue shows the mild-moderate ARDS group, and red represents the moderate-severe ARDS group. In the depicted figure, the moderate-severe ARDS group exhibited higher values for the SOFA score, respiratory rate (RR), positive end expiratory pressure (PEEP), plateau pressure (Pplat), mechanical power (MP), and elastic power (EP), as compared to the mild-moderate ARDS group

A retrospective analysis was conducted on 61,532 hospitalized patients in the MIMIC-III v1.4 database, out of which 5,198 patients had undergone invasive ventilation for ≥ 48 h. Following the exclusion criteria, a final cohort of 403 patients who met the ARDS Berlin diagnostic criteria was identified. Among them, 107 and 296 patients were categorized into the mild-moderate and moderate-severe ARDS groups, respectively. The screening process is illustrated in Fig. 1. The distribution of baseline characteristics and ventilatory variables between mild-moderate ARDS and moderate-severe ARDS are shown in Fig. 2.

The mean age of the 403 ARDS patients was 61 years (IQR 47–75 years), and 215 (53.3%) were male. Within the second 24 h of ventilation, the EP was 16.6 J/min (IQR 13.2–22.5 J/min). The main etiology of ARDS was respiratory disease (67.5%). The mean IMV duration was 7.0 days (IQR 4.2–12.5 days), the mean ICU LOS was 13.0 days (IQR 8.0–21.5 days), and the mean hospital LOS was 20.0 days (IQR 12.0–29 days). Additional details are summarized in Table 1, and details about the respiratory mechanics parameters of the patients in the first 24 h are shown in Additional file 1: Table S2.

**Table 1:** Patient characteristics and outcome parameters

Variables	Elastic power quartiles					P value
	All (*n* = 403)	Q1 (*n* = 101)	Q2 (*n* = 100)	Q3 (*n* = 101)	Q4 (*n* = 101)	
		≤ 13.2	13.2–16.5	16.5–22.5	≥ 22.5	
Age (years)	61 (47–75)	67 (56–83)	64 (51–76)	58 (48–70)	52 (41–65)	< 0.001
Gender (male), n (%)	215 (53.3)	48 (47.5)	48 (48.0)	60 (59.4)	59 (58.4)	0.168
BMI (kg/m^2^)	27.8 (24.0–32.3)	25.0 (22.5–29.3)	28.8 (25.3–33.2)	28.6 (24.4–34.5)	28.2 (25.6–34.3)	< 0.001
PBW (kg)	63.9 (54.7–72.2)	61.0 (53.7–70.8)	61.6 (54.8–70.8)	66.3 (56.9–73.1)	64.3 (57.1–73.1)	0.045
SOFA score	7 (5–10)	6 (4–8)	7 (5–9)	7 (5–10)	8 (5–12)	< 0.001
SAPS II	45 (33–56)	44 (35–55)	45 (36–57)	43 (31–55)	46 (33–58)	0.611
Causes of ARDS, n (%)						
Sepsis	139 (34.5)	24 (23.8)	32 (32.0)	40 (39.6)	43 (42.6)	0.023
Respiratory disease	272 (67.5)	64 (63.4)	69 (69.0)	65 (64.4)	74 (73.3)	0.411
Trauma	20 (5.0)	1 (1.0)	2 (2.0)	10 (9.9)	7 (6.9)	0.013
Others	54 (13.4)	20 (19.8)	9 (9.0)	18 (17.8)	7 (6.9)	0.014
Comorbidities, n (%)						
Coronary artery	50 (12.4)	21 (20.8)	8 (8.0)	11 (10.9)	10 (9.9)	0.028
COPD	25 (6.2)	8 (7.9)	7 (7.0)	4 (4.0)	6 (5.9)	0.681
Chronic liver	5 (1.2)	1 (1.0)	0 (0)	1 (1.0)	3 (3.0)	0.400
Chronic renal	17 (4.2)	2 (2.0)	5 (5.0)	0 (0)	10 (9.9)	< 0.001
Diabetes	95 (23.6)	19 (18.8)	32 (32.0)	23 (22.8)	21 (20.8)	0.128
Stroke	12 (3.0)	3 (3.0)	3 (3.0)	3 (3.0)	3 (3.0)	1.000
Physiological variables in the first 24 h						
PH	7.31 (7.22–7.38)	7.32 (7.26–7.37)	7.32 (7.23–7.37)	7.30 (7.23–7.38)	7.30 (7.22–7.36)	0.311
PCO_2_ (mmHg)	43.0 (37.0–53.0)	42.0 (37.0–47.4)	42.0 (37.8–51.0)	43.0 (37.0–52.0)	43.0 (38.4–54.0)	0.364
Lowest PO_2_/FiO_2_ ratio (mmHg)	120.0 (83.3–155.0)	157.1 (113.9–185.0)	124.5 (97.1–151.2)	115.0 (79.0–138.3)	86.2 (62.0–120.0)	< 0.001
Respiratory mechanics parameters in the second 24 h						
VT (ml/kg PBW)	8.1 (7.0–9.2)	8.0 (6.9–9.1)	8.5 (7.6–9.7)	7.9 (6.9–9.0)	7.7 (6.5–8.9)	0.003
PEEP (cmH_2_O)	8.5 (5.0–12.0)	5.0 (5.0–7.0)	6.9 (5.0–9.0)	10.0 (7.5–12.0)	13.5 (10.0–16.2)	< 0.001
Pplat (cmH_2_O)	22.8 (19.0–27.5)	18.2 (16.5–20.5)	21.8 (19.2–23.8)	23.8 (21.5–27.2)	29.8 (27.0–34.0)	< 0.001
ΔP (cmH_2_O)	14.0 (11.5–17.2)	12.5 (10.0–14.3)	14.6 (12.0–16.6)	13.5 (11.0–17.0)	16.0 (13.5–19.8)	< 0.001
RR (bpm)	22.0 (18.0–25.8)	18.0 (15.8–20.2)	20.9 (17.2–23.6)	24.0 (20.0–26.0)	26.5 (23.0–30.2)	< 0.001
Cst (ml/cmH_2_O)	35.4 (28.2–45.4)	41.1 (33.4–51.5)	37.8 (30.3–51.3)	38.9 (28.3–50.1)	33.1 (26.0–39.7)	< 0.001
MP (J/min)	22.6 (17.5–28.8)	14.8 (12.4–17.4)	19.8 (17.9–23.5)	24.9 (21.6–27.5)	32.3 (28.8–39.2)	< 0.001
EP (J/min)	16.6 (13.2–22.5)	11.1 (9.7–12.4)	14.8 (13.9–15.8)	19.2 (18.1–20.8)	27.6 (24.0–30.6)	< 0.001
FiO_2_ (%)	55 (45–71)	45 (40–55)	50 (45–55)	60 (50–75)	70 (55–80)	< 0.001
Clinical outcomes (days)						
IMV duration	7.0 (4.2–12.5)	5.9 (3.4–8.9)	5.3 (3.6–9.6)	7.2 (4.6–11.5)	10.3 (5.9–16.6)	< 0.001
ICU LOS	13.0 (8.0–21.5)	10.0 (7.0–16.0)	11.0 (8.0–19.2)	13.0 (8.0–22.0)	17.0 (12.0–27.0)	< 0.001
Hospital LOS	20.0 (12.0–29.0)	18.0 (11.0–26.0)	17.0 (11.0–26.0)	20.0 (12.0–31.0)	21.0 (16.0–36.0)	0.013

*BMI* body mass index, *PBW* predicted body weight, *SOFA* sequential organ failure assessment, *SAPS II* simplified acute physiology score II, *COPD* chronic obstructive pulmonary disease, *PCO*_*2*_ partial pressure of carbon dioxide, *PaO*_*2*_*/FiO*_*2*_ oxygenation index i.e. arterial partial pressure of oxygen (PaO_2_) divided by the inspired oxygen fraction (FiO_2_), *VT* tidal volume, *PEEP* positive end expiratory pressure, *Pplat* plateau pressure, *ΔP* driving pressure, *RR* respiratory rate, *Cst* static lung compliance, *MP* mechanical power, *EP* elastic power, *IMV* invasive mechanical ventilation, *LOS* length of stay

Q1, Q2, Q3, and Q4 are quartiles of the elastic power (EP)

### Relationship between EP and the severity of ARDS

**Fig. 3:**
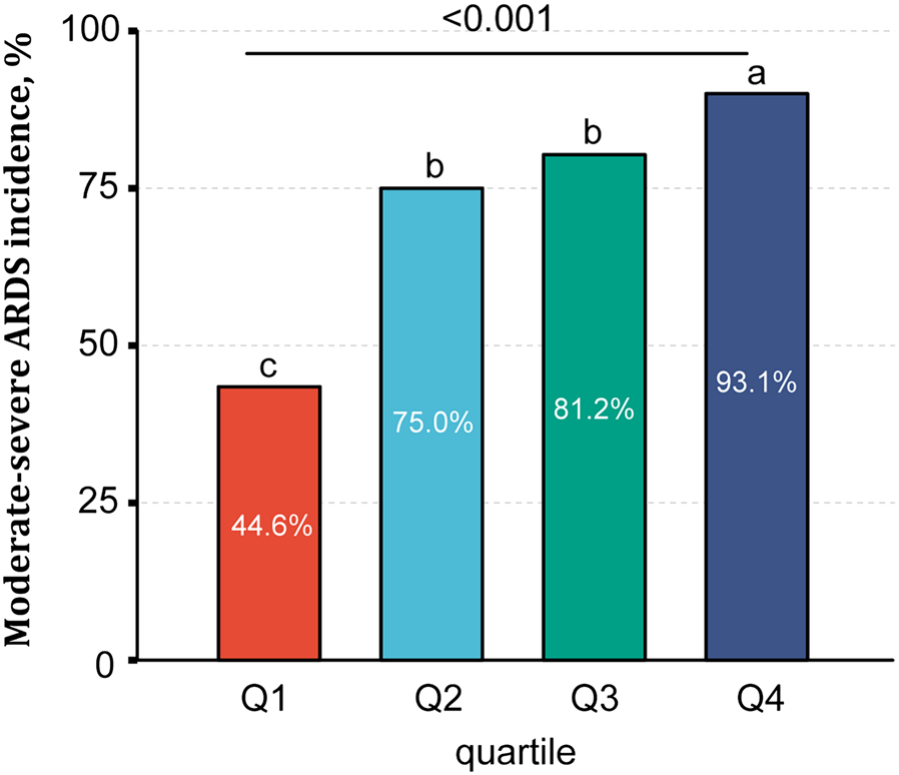
Moderate-severe ARDS incidence as expressed in elastic power (EP) quartile. Corresponding quartile cut-of values are shown in Table 1. Moderate-severe ARDS incidence is higher in Q4 compared to Q1. Different letters represent significant differences between different groups (*P* < 0.05). Q1, Q2, Q3, and Q4 are quartiles of the EP

The total incidence of moderate-severe ARDS was 73.4% (296/403). The incidence is higher in the fourth EP quartile ( 93.1%) as compared to the first EP quartile (44.6%) (p < 0.001, Fig. 3). Spearman correlation analysis indicated that EP was significantly negatively correlated with P/F ratio ( R = -0.46, p < 0.001, Additional file: Figure S2).

The results from univariate analysis demonstrated that age, SOFA score, PCO_2_, PEEP, Pplat, RR, MP, EP, and FiO_2_ were significantly associated with the incidence of moderate-severe ARDS (Table 2). In logistic regression analysis with EP expressed as a continuous variable, EP was associated with moderate-severe ARDS (odds ratio [OR] 1.21, 95% confidence interval [CI] 1.15–1.28, p < 0.001, Table 3, Model 1). This association remained independent after adjustment for confounders, with an OR of 1.20 (95%CI 1.13–1.27, p = 0.001, Table 3, model 3). In multivariable logistic regression analyses with EP expressed in quartiles, there was a 14.12 times increased risk of moderate-severe ARDS incidence in the highest quartile when compared with that in the lowest quartile (OR 14.12, 95% CI 5.69–35.07, p = 0.016), independent of potential confounders (Table 3, Model 3).

**Table 2:** Univariate logistic regression analysis of risk factors for moderate-severe ARDS

Variables	OR (95% CI)	P value	Variables	OR (95% CI)	P value
Age (years)	0.98 (0.97–0.99)	0.005	Chronic renal	1.18 (0.38–3.71)	0.773
Gender (male)	1.00 (0.64–1.56)	0.985	Diabetes	0.95 (0.56–1.59)	0.836
BMI (kg/m^2^)	1.03 (1.00–1.07)	0.071	Respiratory mechanics parameters		
PBW (kg)	1.00 (0.98–1.03)	0.653	VT (ml/kg PBW)	1.02 (0.89–1.17)	0.739
SOFA score	1.11 (1.04–1.18)	0.003	PEEP (cmH_2_O)	1.31 (1.21–1.43)	< 0.001
SAPS II	1.00 (0.98–1.01)	0.790	Pplat (cmH_2_O)	1.15 (1.1–1.21)	< 0.001
Causes of ARDS			ΔP (cmH_2_O)	1.04 (0.99–1.10)	0.096
Sepsis	1.18 (0.74–1.89)	0.491	RR (bpm)	1.11 (1.06–1.17)	< 0.001
Respiratory disease	1.07 (0.67–1.72)	0.769	Cst (ml/cmH_2_O)	0.99 (0.98–1.01)	0.445
Trauma	3.4 (0.78–14.9)	0.105	MP (J/min)	1.15 (1.11–1.20)	< 0.001
Others	0.62 (0.34–1.14)	0.125	EP (J/min)	1.21 (1.15–1.28)	< 0.001
Comorbidities			FiO_2_ (%)	1.06 (1.04–1.08)	< 0.001
Coronary artery	0.66 (0.35–1.25)	0.205	Physiological variables in the first 24 h		
COPD	1.48 (0.54–4.04)	0.446	PH	0.21 (0.03–1.64)	0.135
Chronic liver	0.54 (0.09–3.26)	0.500	PCO_2_ (mmHg)	1.04 (1.02–1.06)	< 0.001

*ARDS* acute respiratory distresssyndrome, *BMI* body mass index, *PBW* predicted body weight, *SOFA* sequential organ failure assessment, *SAPS II* simplified acute physiology score II, *COPD* chronic obstructive pulmonary disease, *PCO*_*2*_ partial pressure of carbon dioxide, *VT* tidal volume, *PEEP* positive end expiratory pressure, *Pplat* plateau pressure, *ΔP* driving pressure, *RR* respiratory rate, *Cst* static lung compliance, *MP* mechanical power, *EP* elastic power, *FiO*_*2*_ inspired oxygen fraction

**Table 3:** Multivariable-adjust ORs and 95%CI of the elastic power quartiles associated with moderate-severe ARDS

Variables	Unadjusted		Model 1		Model 2		Model 3	
	OR (95% CI)	P value	OR (95% CI)	P value	OR (95% CI)	P value	OR (95% CI)	P value
EP (J/min)	1.21 (1.15–1.28)	< 0.001	1.21 (1.15–1.28)	< 0.001	1.20 (1.14–1.28)	< 0.001	1.20 (1.13–1.27)	0.001
Q1 (≤ 13.2)	1 (Ref)		1 (Ref)		1 (Ref)		1 (Ref)	
Q2 (13.2–16.5)	3.73 (2.05–6.80)	< 0.001	3.75 (2.03–6.96)	< 0.001	3.58 (1.92–6.65)	< 0.001	3.57 (1.89–6.75)	0.001
Q3 (16.5–22.5)	5.37 (2.85–10.13)	< 0.001	5.20 (2.69–10.08)	< 0.001	4.85 (2.48–9.47)	< 0.001	4.98 (2.52–9.86)	0.021
Q4 (≥ 22.5)	16.71 (7.05–39.58)	< 0.001	15.88 (6.52–38.65)	< 0.001	14.09 (5.72–34.72)	< 0.001	14.12 (5.69–35.07)	0.016
Trend test	2.44 (1.92–3.12)	< 0.001	2.39 (1.85–3.09)	< 0.001	2.30 (1.77–2.98)	< 0.001	2.31 (1.77–3.01)	0.009

*OR* odds ratio, *CI* confidence interval, *EP* elastic power, *Ref* reference

Q1, Q2, Q3, and Q4 are quartiles of the EP

Model 1 was adjusted for age and BMI

Model 2 was adjusted for Model 1 + SOFA score

Model 3 was adjusted for Model 2 + MBP, and PCO_2_

**Fig. 4:**
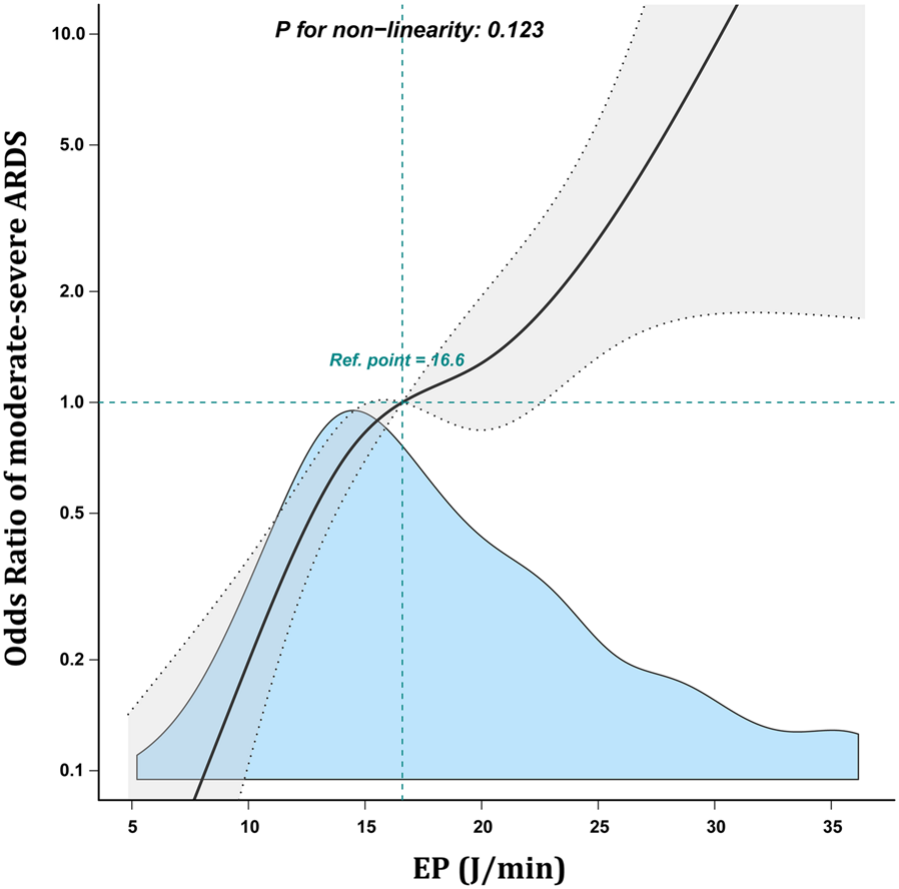
Association between elastic power (EP) with moderate-severe ARDS. Solid line represents the best fit linear regression, and dashed lines represent a 95% confidence interval. Odd ratios (ORs) were adjusted for age, BMI, SOFA score, MBP and PCO_2_. The blue region represents the density map of the frequency distribution of moderate-severe ARDS

A multivariable adjusted restricted cubic spline for the association is shown in Fig. 4. A linear relationship between EP and the incidence of moderate-severe ARDS was observed (p value for nonlinearity = 0.123).

### Subgroup analyses by adjusted potential effect confounders

**Fig. 5:**
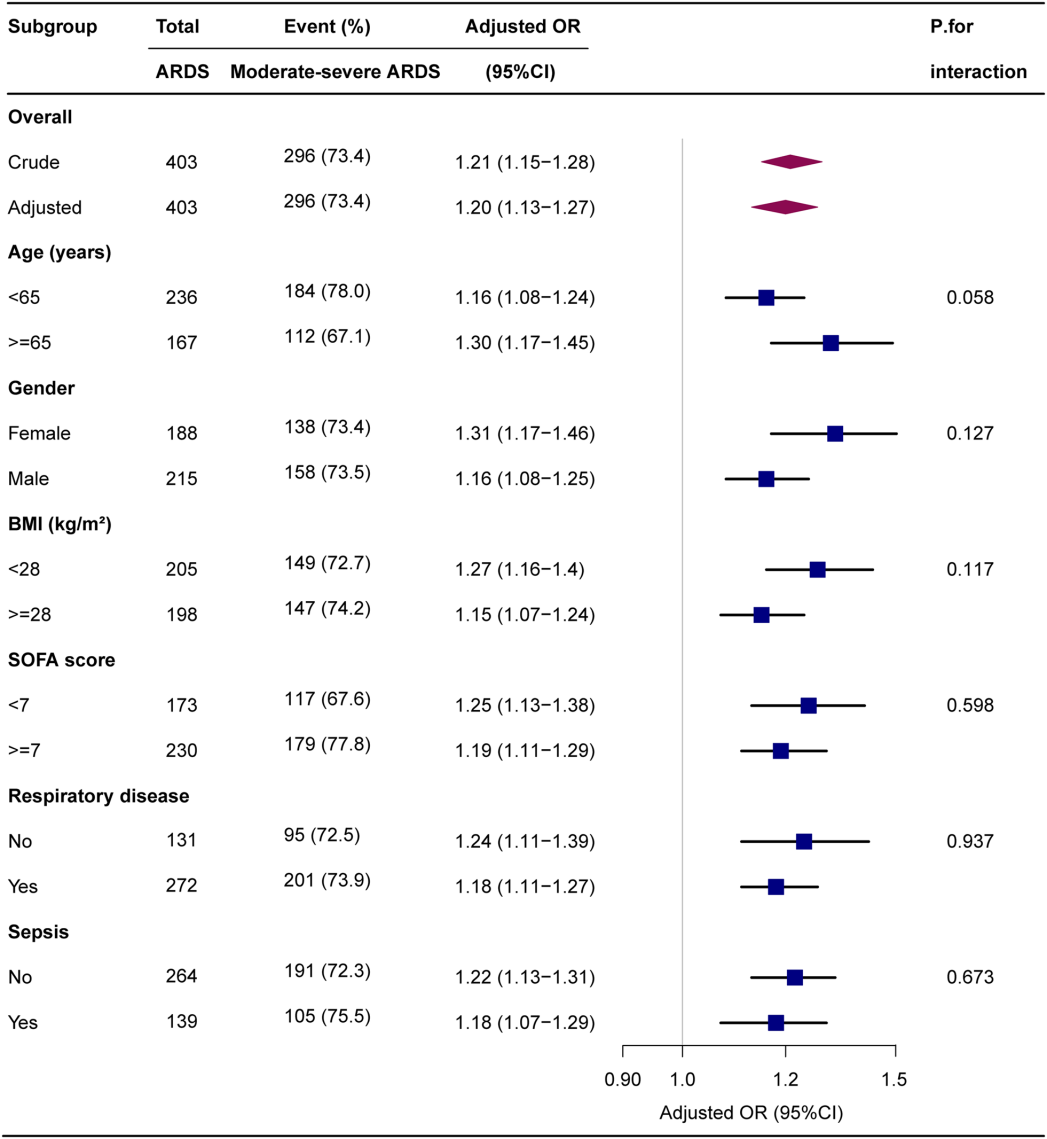
Subgroup analyses of the elastic power (EP) and moderate-severe ARDS. Each stratification was adjusted for age, BMI, SOFA score, MBP and PCO_2_ except the stratification factor itself. Diamonds indicate overall odds ratios (ORs), with outer points of the diamonds indicating 95% CIs. Squares indicate ORs, with horizontal lines indicating 95%CIs

Subgroup analyses were performed to assess the impact of EP (per 1 unit increment) on moderate-severe ARDS in distinct subgroups (Fig. 5). No significant interactions were found in any subgroups after stratifying by age, gender, BMI, SOFA score, respiratory disease, and sepsis (Fig. 5). When EP was analyzed as quartiles, subgroup analysis showed that the relationship remained robust and reliable (Additional file 1: Figure S3).

### Sensitivity analysis and additional analyses

Sensitivity analyses were conducted to assess the robustness of the results. First, a multivariate logistic regression model was performed in cases with complete data (Additional file 1: Table S3). In addition, the association between EP and the incidence of moderate-severe ARDS was assessed using data from the first 24 h in an additional sensitivity analysis (Additional file 1: Table S4). Notably, similar associations were found in both these sensitivity analyses (additional details are provided in the Supplementary Materials). Additional analyses were shown in Additional file 1: Table S5.

## Discussion

The novel findings of this study can be summarized as follows: First, the effect of EP was found dependent on the severity of ARDS being higher with increasing severity. Second, EP displayed a higher area under the receiver operating characteristic curve than other individual variables in predicting ARDS severity. These results support the hypothesis that EP can be used as a new indicator for the stratification of ARDS and assessment of ARDS severity from the perspective of respiratory mechanics. Overall, these findings have important implications for the clinical management of ARDS patients and may lead to the development of targeted and effective interventions.

The essence of mechanical ventilation in ARDS patients is the mechanical process whereby breathing power overcomes the respiratory system resistance and drives the gas delivery to the body. Therefore, an in-depth understanding of the respiratory mechanics characteristics of ARDS patients during mechanical ventilation is of paramount clinical importance for evaluating the disease severity and predicting the prognosis of the patient [14, 15]. Various ventilator variables have been studied in previous studies [16]. Early studies have shown that low-VT ventilation is not only beneficial for ARDS patients but also for individuals with healthy lungs [17]. In fact, low VT is one of the components of protective ventilation, along with PEEP, Pplat, RR, and Ppeak. ΔP, which is calculated by subtracting PEEP from Pplat, has received increased attention in recent years because it considers Cst. Studies have demonstrated that a high ΔP is strongly associated with high mortality [18]. However, ΔP is only one of several energy parameters involved in acting on the lung parenchyma; Cst, Vt, flow rate, and RR are also key factors affecting lung injury [19]. As a relatively more comprehensive and integrated parameter of lung energy loading, As an important component of MP, EP is essential to comprehend the biomechanical properties of the interaction between the ventilator and the lung tissue in ARDS patients subjected to mechanical ventilation, and to evaluate the severity of ARDS [20].

Neto et al. [7] have analyzed 8,207 patients who received invasive ventilation for ≥ 48 h and have found that the MP in the second 24 h of ventilation is independently associated with the increased mortality of critically ill patients, fewer ventilator-free days, and lower survival on day 28. Concordantly, Umer et al. [21] have reported that cumulative exposure to high intensities of mechanical ventilation is harmful and that a significant increase in the hazard of death is associated with each daily increment in ΔP and MP. In contrast, Coppola et al. [22] have reported that MP resulting from airway pressure and that from transpulmonary pressure are not related to the outcome of ARDS patients. Costa et al. [6] conducted a study based on 4,549 ARDS patients and found that the novel combined ventilator variable [(4 × DP) + RR] was signifcantly associated with mortality and was comparable as MP. Our analysis indicates that while [4ΔP + RR] showed a significant association with the occurrence of moderate-severe ARDS [OR (95% CI) 1.02 (1.00–1.03)], its discriminatory ability for moderate-severe ARDS was comparatively weaker when compared with EP (Additional file 1: Table S6 and Figure S4). From an integrative physiological and pathophysiological perspective, ARDS is characterized by several acute pathological changes, including a significant reduction in pulmonary compliance, ventilation capacity, and heterogeneous changes in the lungs. Although increased airway resistance is not a hallmark feature of ARDS, it may be pronounced in patients with underlying conditions, such as COPD or asthma [23]. Therefore, as we found, when compared with other parameters, EP correlates more with the disease severity in ARDS patients. In multivariable logistic regression analyses with EP expressed in quartiles, there was a 14.12 times increased risk of moderate-severe ARDS incidence in the highest quartile compared with that in the lowest quartile (OR 14.12, 95% CI 5.69–35.07), and there was a linear relationship between EP and incidence of moderate-severe ARDS (p value for nonlinearity = 0.123). These results strongly suggest that EP is a more comprehensive respiratory mechanics index than the other indices for assessing ARDS severity. Consequently, EP may help clinicians in precisely grading ARDS severity in the future.

During mechanical ventilation in ARDS, lung injury arises from the interaction between the total energy delivered to the lung tissue by the ventilator and the anatomical-pathological characteristics of the lung tissue itself. Cressoni et al. [24] have found that total MP contributes to VILI. Therefore, the parameters of MP components may be set differently, but the effects will be similar as long as the VILI threshold of the MP is exceeded. However, during ventilator delivery, the energy consumed by the airflow is mainly used to overcome airway resistance, which is difficult to correlate with alveolar injury, as the energy carried by the airflow itself does not necessarily lead directly to lung injury [6]. Identification of the specific energy that acts on the lung parenchyma will help clinicians assess the true energy that causes lung injury. The classic ARDS lung-protective ventilation strategy highlights that airway resistance and peak airway pressure do not significantly correlate with lung injury. Instead, the combined effect of Pplat, ΔP, VT, and altered Cst are the parameters that clinicians should focus to prevent lung tissue injury[19, 25], and the combined effect of these parameters is expressed as EP. EP and lung injury are causally related to each other. Therefore, limiting EP to a relatively safe range may help to prevent further exacerbation of lung injury.

Our findings suggest also that the higher RR could be another important component of VILI, rather than just multiplication factor to contribute to total EP or MP. It may also be due to improper settings or excessively strong spontaneous breathing, leading to a higher RR, which may cause or aggravate lung damage. Although still susceptible to residual confounding, these findings suggest that not only is the degree of stress/strain per breath important (captured by ΔP), but also how often the stress/strain is repeated (captured by RR).

There are several limitations to this study that should be mentioned. First, as with any regression analysis, residual confounders may still exist. Through subgroup and sensitivity analyses, we attempted to adjust for possible confounders and minimize the influence of factors that may lead to outcome bias. Second, because of the cross-sectional nature of the study, we could not determine the temporal association between EP and the severity of ARDS. However, we conducted sensitivity analyses at various time points to investigate the stability of the associations. Third, as both Pplat and PEEP are an important part of equation to calculate the EP. Using exact formula for Pplat, we need to apply end-inspiratory hold, which was not performed given the retrospectively analysed data. Similarly, end-expiratory hold was not performed to verify intrinsic PEEP (and therefore PEEPtot). There was no guarantee that the parameters were collected under standard conditions without spontaneous breathing and sufficient sedation. We must recognise that prospective trial with strict protocol would have been a much better study design. Fourth, the association between EP normalized to functional lung size and ARDS severity needs further investigation.

## Conclusion

EP is significantly associated with ARDS severity and should be carefully monitored in patients undergoing mechanical ventilation. IIn addition, it may be used for grading ARDS severity. However, further experimental trials are needed to investigate whether adjusting ventilator variables according to EP will significantly improve clinical outcomes.

### Supplementary Information

**Additional fle 1: Table S1.** Percentage of missing data in the variables of interest at baseline; **Table S2.** Respiratory mechanics parameters of the study population according to elastic power quartiles in the first 24 h; **Table S3.** Association between elastic power and moderate-severe ARDS in complete cases; **Table S4.** Association between elastic power and moderate-severe ARDS in the first 24 h; **Table S5.** Capability of related parameters in predicting moderate-severe ARDS occurrence in ARDS patients; **Table S6.** Factors associated with moderate-severe ARDS by of binary logistic regression analysis; **Figure S1.** The relationship between elastic power and moderate-severe ARDS according to basic features; **Figure S2.** Comparision of Receiver operating characteristic curves for [4ΔP + RR] and EP.

## Data Availability

The datasets presented in the current study are available in the MIMIC III database (https://www.physionet.org/content/mimiciii/1.4/).

## Abbreviations

ARDS: Acute respiratory distresssyndrome
BMI: Body mass index
PBW: Predicted body weight
SOFA: Sequential organ failure assessment
SAPS II: Simplified acute physiology score II
COPD: Chronic obstructive pulmonary disease
RRT: Renal replacement therapy
HR: Heart rate
MBP: Mean blood pressure
PCO_2_: Partial pressure of carbon dioxide
SPO_2_: Pulse oximetry
VT: Tidal volume
PEEP: Positive end expiratory pressure
Pplat: Plateau pressure
ΔP: Driving pressure
RR: Respiratory rate
Cst: Static lung compliance
MP: Mechanical power
EP: Elastic power
FiO_2_: Inspired oxygen fraction
IMV: Invasive mechanical ventilation
LOS: Length of stay
