# Supplementary material for: Association of elastic power in mechanical ventilation with the severity of acute respiratory distress syndrome: a retrospective study
Source: Eur J Med Res. 2024 Jan 3;29:5. doi: 10.1186/s40001-023-01577-7 (PMC10763103; doi:10.1186/s40001-023-01577-7)
Supplement: Supplementary file 1 — Additional fle 1: Table S1. Percentage of missing data in the variables of interest at baseline; Table S2. Respiratory mechanics parameters of the study population according to elastic power quartiles in the first 24 h; Table S3. Association between elastic power and moderate-severe ARDS in complete cases; Table S4. Association between elastic power and moderate-severe ARDS in the first 24 h; Table S5. Capability of related parameters in predicting moderate-severe ARDS occurrence in ARDS patients; Table S6. Factors associated with moderate-severe ARDS by of binary logistic regression analysis; Figure S1. The relationship between elastic power and moderate-severe ARDS according to basic features; Figure S2. Comparision of Receiver operating characteristic curves for [4ΔP + RR] and EP. [file 40001_2023_1577_MOESM1_ESM.docx]

**Association of Elastic Power in Mechanical Ventilation with the Severity of Acute Respiratory Distress Syndrome: A Retrospective Study**

**Additional information**

1. **Glossary of terms (Page 2)**
2. **Supplementary methods (Page 3)**
3. **Supplementary results (Page 3)**
4. **Table S1 Percentage of missing data in the variables of interest at baseline (Page 5)**
5. **Table S2 Respiratory mechanics parameters of the study population according to elastic power quartiles in the first 24h (Page 6)**
6. **Table S3 Association between elastic power and moderate-severe ARDS in complete cases (Page 7)**
7. **Table S4 Association between elastic power and moderate-severe ARDS in the first 24h (Page 8)**
8. **Table S5 Capability of related parameters in predicting moderate-severe ARDS occurrence in ARDS patients (Page 9)**
9. **Table S6 Factors associated with moderate-severe ARDS by of binary logistic regression analysis (Page 9)**
10. **Fig. S1 Graphical representation of the equation of power(Page 10)**
11. **Fig. S2 Correlation Between EP and PaO_2_/FiO_2_ ratio in ARDS Patients (Page 11)**
12. **Fig. S3 The relationship between elastic power and moderate-severe ARDS according to basic features (Page 12)**
13. **Fig. S4 Comparision of Receiver operating characteristic curves for [4ΔP+RR] and EP (Page 13)**

| **Glossary of terms** |  |  |
| --- | --- | --- |
| **Medical term** |  | **Explanation** |
| **MIMIC-IV** |  | Medical information mart for intensive care IV |
| **ICU** |  | Intensive care unit |
| **FiO_2_/FiO_2_** |  | Oxygenation index, i.e. arterial partial pressure of oxygen (PaO_2_) divided by the inspired oxygen concentration (FiO_2_) |
| **BMI** |  | Body mass index |
| **PBW** |  | Predicted body weight (PBW) was calculated as follows:  **PBW (male) = 50 + 0.91 [height (cm) – 152.4]**  **PBW (female) = 45.5 + 0.91 [height (cm) – 152.4]** |
| **SAPS II** |  | Simplified acute physiology score II |
| **SOFA** |  | Sequential organ failure assessment |
| **SAPS II** |  | Simplified acute physiology score II |
| **COPD** |  | Chronic obstructive pulmonary disease |
| **RRT** |  | Renal replacement therapy |
| **PCO_2_** |  | Partial pressure of carbon dioxide |
| **SPO_2_** |  | Pulse oximetry |
| **VT** |  | Tidal volume |
| **RR** |  | Respiratory rate |
| **Positive end expiratory pressure (PEEP)** |  | Positive end-expiratory pressure (PEEP) as recorded was the external or applied PEEP, not the total PEEP, or intrinsic PEEP. |
| **Plateau pressure (Pplat)** |  | The plateau pressure (Pplat) was measured during an inspiratory pause on the ventilator. |
| **Peak inspiratory pressure (Ppeak)** |  | Peak inspiratory pressure (Ppeak) should be obtained while the patient is relaxed, not coughing or moving in bed. |
| **Driving pressure (ΔP)** |  | Driving pressure (ΔP) in the ventilation mode was calculated using Pplat and PEEP:   Δ**P (cmH_2_O) = Pplat ‒ PEEP** |
| **Static lung compliance (Cst)** |  | Static lung compliance (Cst) refers to the change in lung volume caused by a unit pressure change, reflecting the compliance of the respiratory system and was calculated using VT and ΔP: **Cst = V_T_ /ΔP** |
| **Mechanical power (MP)** |  | Mechanical power (MP) was calculated according to Gattinoni's simplified mechanical power equation as follows:   **MP (J/min) = 0.098×VT×RR×(Ppeak – ½ΔP)** |
| **Elastic power (EP)** |  | Elastic power (EP) refers to the energy done by the ventilator to overcome the elastic resistance of the respiratory system in one minute and was calculated as follows:  **EP (J/min) = 0.098×VT×RR×½ (PEEP+Pplat)** |

**Additional methods**

Approach to missing Data

Missing data were summarized in Table S1. We assumed that missing data was “missing at random”. Multiple imputation by chained equations (MICE) was then conducted to impute covariates. In total we generated 10 different  data sets and used ‘mice’ package in R studio to impute the data (https://cran.rproject.org/web/packages/ mice).

**Additional results**

Subgroup analyses

We further performed stratified analysis according to Elastic Power (EP) quariles. No signiﬁcant interactions were found in any subgroups after stratifying by age, gender, BMI, SOFA score, respiratory disease and sepsis (Fig. S1). There was similar association between EP and moderate-severe ARDS.

Sensitivity analyses

As sensitivity analyses, we repeated the analyses in cases with only complete data (67.5%; n=272), without imputation of missing data. Those with complete data did similarly not differ from those with imputation of missing data. This association between EP and moderate-severe ARDS was unchanged in multivariable models (OR 1.17, 95%CI 1.10-1.25, *P*<0.001; Table S2, model 3). After full adjustments, these associations remained significant when EP was treated as a categorical variable (*P*<0.01; Table S2, model 3).

Given the cross-sectional study design could not consider the time effect on association between EP and moderate-severe ARDS, we have therefore conducted a sensitivity analysis with the data in the first 24h (Table S3, Table S4). There was an unchanged independent association of EP with moderate-severe ARDS (OR 1.11, 95%CI 1.06-1.15, *P*<0.001; Table S4, model 3). The association between EP and moderate-severe ARDS did not markedly change when EP was expressed as quartiles. A 9-fold increased risk of moderate-severe ARDS was found for high (fourth quartile) versus low (first quartile) EP levels (OR 9.42, 95% CI 3.60-24.61, *P*<0.001; Table S4, model 3).

Predictive value of related parameters

We assessed the predictive value for moderate-severe ARDS by EP, Pplat, ΔP, Cst and MP using the ROC curves analysis (Table S5). EP had an area under ROC curve (AUC) of 0.77 (95%CI 0.72-0.82, *P*<0.001) for the prediction of moderate-severe ARDS. The cut-off value of EP was 14.6 J/min with sensitivity of 75% and specificity of 66%. The AUC for the MP was 0.75 (95%CI 0.70-0.81, *P*<0.001) for moderate-severe ARDS. The cut-off value of MP was 18.1 J/min with sensitivity of 81% and specificity of 59%. The AUC for the Pplat, ΔP and Cst were 0.70 (95%CI 0.65-0.76, *P*<0.001), 0.55 (95%CI 0.49-0.62, *P*=0.106) and 0.55 (95%CI 0.49-0.61, *P*=0.134), respectly. EP has a better behavior in the prediction of ARDS severity than the other respiratory mechanics parameters in terms of AUC (Table S5).

**Table S1** **Percentage of missing data in the variables of interest at baseline**

| **Variables** | **n (%)** |
| --- | --- |
| Age (years) | 0 (0%) |
| Gender (male) | 0 (0%) |
| BMI (kg/m^2^) | 74 (18.4%) |
| PBW (kg/m^2^) | 74 (18.4%) |
| SOFA score | 0 (%) |
| SAPS II | 0 (%) |
| SPO_2_ (%) | 32 (7.9%) |
| PH | 49 (12.2%) |
| PaCO_2_ (mmHg) | 49 (12.2%) |
| Lowest PO_2_/FiO_2_ ratio (mmHg) | 0 (%) |
| VT (ml) | 0 (%) |
| PEEP (cmH_2_O) | 0 (%) |
| Pplat (cmH_2_O) | 0 (%) |
| ΔP (cmH_2_O) | 0 (%) |
| RR (bpm) | 0 (%) |
| Cst (ml/cmH_2_O) | 0 (%) |
| MP (J/min) | 0 (%) |
| EP (J/min×cmH_2_O) | 0 (%) |
| FiO_2_ (%) | 0 (%) |

BMI, body mass index; PBW, predicted body weight; SOFA, sequential organ failure assessment; SAPS II, simplified acute physiology score II; SPO_2_, pulse oximetry; PaCO_2_, arterial partial pressure of carbon dioxide; PaO_2_/FiO_2_, oxygenation indexarterial, (i.e., PaO_2_ divided by the FiO_2_); VT, tidal volume; PEEP, positive end expiratory pressure; Pplat, plateau pressure; ΔP, driving pressure; RR, respiratory rate; Cst, static lung compliance; MP, mechanical power; EP, elastic power; FiO_2_, inspired oxygen concentration.

**Table S2 Respiratory mechanics parameters of the study population according to elastic power quartiles in the first 24h**

| **Variables** | **All** | **Q1** | **Q2** | **Q3** | **Q4** | ***p*** |  |
| --- | --- | --- | --- | --- | --- | --- | --- |
|  | **(n=403)** | **(n=101)** | **(n=100)** | **(n=101)** | **(n=101)** | **value** |  |
| VT (ml/kg PBW) | 8.3 (7.5-9.3) | 8.2 (7.6-9.0) | 8.6 (7.7-9.5) | 8.2 (7.4-9.4) | 8.3 (7.3-9.4) | 0.330 |  |
| PEEP (cmH_2_O) | 8.0 (5.0-10.8) | 5.0 (5.0-7.5) | 6.8 (5.0-8.8) | 8.8 (6.2-11.0) | 12.0 (9.5-15.0) | < 0.001 |  |
| Pplat (cmH_2_O) | 23.5 (20.0-28.0) | 19.5 (17.0-22.0) | 22.7 (20.7-25.6) | 24.0 (21.2-28.5) | 29.0 (26.0-32.5) | < 0.001 |  |
| ΔP (cmH_2_O) | 14.8 (12.2-18.1) | 13.0 (11.0-15.8) | 15.0 (12.3-18.0) | 15.0 (12.0-18.2) | 16.0 (13.5-19.8) | < 0.001 |  |
| RR (bpm) | 22.2 (18.6-26.0) | 18.8 (16.2-22.0) | 21.8 (18.0-24.5) | 24.0 (20.0-27.0) | 25.2 (21.8-28.2) | < 0.001 |  |
| Cst (ml/cmH_2_O) | 36.9 (30.0-47.3) | 41.4 (32.6-50.3) | 36.8 (31.5-44.5) | 37.8 (29.4-49.5) | 33.8 (28.9-39.2) | 0.002 |  |
| MP (J/min) | 24.0 (18.0-31.1) | 17.3 (13.7-22.2) | 22.1 (18.0-28.1) | 26.4 (20.9-31.1) | 32.8 (26.9-38.4) | < 0.001 |  |
| EP (J/min) | 18.5 (13.9-24.4) | 12.3 (9.6-15.4) | 16.4 (14.3-20.1) | 21.8 (15.8-24.6) | 26.6 (22.2-31.1) | < 0.001 |  |
| FiO_2_ (%) | 70 (50-75) | 55 (45-70) | 63 (50-75) | 70 (60-75) | 75 (65-80) | < 0.001 |  |

VT, tidal volume; PEEP, positive end expiratory pressure; Pplat, plateau pressure; ΔP, driving pressure; RR, respiratory rate; Cst, static lung compliance; MP, mechanical power; EP, elastic power; FiO_2_: FiO_2_, inspired oxygen concentration; Q1, Q2, Q3, and Q4 are quartiles of the elastic power (EP).

| **Table S3 Association between elastic power and moderate-severe ARDS** **in complete cases** | | | | | | | | | | | | |
| --- | --- | --- | --- | --- | --- | --- | --- | --- | --- | --- | --- | --- |
| **Variables** | **Unadjusted** | |  | **Model 1** | |  | **Model 2** | |  | **Model 3** | | |
|  | **OR (95% CI)** | ***p* value** |  | **OR (95% CI)** | ***p* value** |  | **OR (95% CI)** | ***p* value** | |  | **OR (95% CI)** | ***p* value** |
| EP (J/min) | 1.18 (1.11-1.26) | <0.001 |  | 1.18 (1.11-1.26) | <0.001 |  | 1.18 (1.10-1.26) | <0.001 | |  | 1.17 (1.10-1.25) | <0.001 |
| Q1 (n=68, ≤13.8) | 1 (Ref) |  |  | 1 (Ref) |  |  | 1 (Ref) |  | |  | 1 (Ref) |  |
| Q2 (n=68, 13.8-17.9) | 4.23 (1.96-9.13) | <0.001 |  | 4.41 (2.00-9.75) | <0.001 |  | 4.27 (1.92-9.49) | <0.001 | |  | 4.76 (2.09-10.85) | <0.001 |
| Q3 (n=68, 17.9-23.8) | 3.53 (1.68-7.44) | 0.001 |  | 3.39 (1.54-7.44) | 0.002 |  | 3.20 (1.43-7.17) | 0.005 | |  | 3.39 (10.47-7.78) | 0.004 |
| Q4 (n=68, ≥23.8) | 33.00 (7.48-145.68) | <0.001 |  | 30.70 (6.69-140.83) | <0.001 |  | 28.55 (6.13-133.01) | <0.001 | |  | 30.29 (6.44-142.54) | <0.001 |
| Trend test | 2.42 (1.77-3.29) | <0.001 |  | 2.36 (1.69-3.29) | <0.001 |  | 2.30 (1.64-3.23) | <0.001 | |  | 2.35 (1.66-3.33) | <0.001 |
| OR, odds ratio; CI, confidence interval; EP, elastic power; Ref: reference; Q1, Q2, Q3, and Q4 are quartiles of the elastic power (EP).  Model 1 was adjusted for age and BMI.  Model 2 was adjusted for Model 1+SOFA score.  Model 3 was adjusted for Model 2+ MBP, and PCO_2_. | | | | | | | | | | | | |

| **Table S4 Association between elastic power and moderate-severe ARDS in the first 24h** | | | | | | | | | | | | |
| --- | --- | --- | --- | --- | --- | --- | --- | --- | --- | --- | --- | --- |
| **Variables** | **Unadjusted** | |  | **Model 1** | |  | **Model 2** | |  | **Model 3** | | |
|  | **OR (95% CI)** | ***p* value** |  | **OR (95% CI)** | ***p* value** |  | **OR (95% CI)** | ***p* value** | |  | **OR (95% CI)** | ***p* value** |
| EP (J/min) | 1.12 (1.08-1.17) | <0.001 |  | 1.12 (1.07-1.17) | <0.001 |  | 1.11 (1.06-1.16) | <0.001 | |  | 1.11 (1.06-1.15) | <0.001 |
| Q1 (n=101, ≤13.9) | 1 (Ref) |  |  | 1 (Ref) |  |  | 1 (Ref) |  | |  | 1 (Ref) |  |
| Q2 (n=100, 13.9-18.4) | 1.70 (0.96-3.01) | 0.070 |  | 1.63 (0.92-2.91) | 0.096 |  | 1.45 (0.80-2.64) | 0.218 | |  | 1.44 (0.79-2.64) | 0.235 |
| Q3 (n=101, 18.4-24.4) | 3.00 (1.63-5.55) | <0.001 |  | 2.84 (1.52-5.28) | 0.001 |  | 2.59 (1.37-4.87) | 0.003 | |  | 2.43 (1.28-4.63) | 0.007 |
| Q4 (n=101, ≥24.4) | 13.24 (5.31-33.01) | <0.001 |  | 11.81 (4.62-30.21) | <0.001 |  | 9.92 (3.81-25.81) | <0.001 | |  | 9.42 (3.60-24.61) | <0.001 |
| Trend test | 2.08 (1.66-2.62) | <0.001 |  | 2.01 (1.58-2.54) | <0.001 |  | 1.91 (1.50-2.43) | <0.001 | |  | 1.87 (1.46-2.39) | <0.001 |
| OR, odds ratio; CI, confidence interval; EP, elastic power; Ref: reference; Q1, Q2, Q3, and Q4 are quartiles of the elastic power (EP).  Model 1 was adjusted for age and BMI.  Model 2 was adjusted for Model 1+SOFA score.  Model 3 was adjusted for Model 2+ MBP, and PCO_2_. | | | | | | | | | | | | |

**Table S5 Capability of related parameters in predicting moderate-severe ARDS occurrence**

**in ARDS patients**

| **Variables** | **AUC (95%CI)** | ***p* value** | **Thresholds** | **Sensitivity** | **Specificity** |
| --- | --- | --- | --- | --- | --- |
| Pplat (cmH_2_O) | 0.70 (0.65-0.76) | <0.001 | >22.1 | 0.57 | 0.72 |
| ΔP (cmH_2_O) | 0.55 (0.49-0.62) | 0.106 | >13.5 | 0.58 | 0.52 |
| Cst (ml/cmH_2_O) | 0.55 (0.49-0.61) | 0.134 | <41.2 | 0.55 | 0.58 |
| MP (J/min) | 0.75 (0.70-0.81) | <0.001 | >18.1 | 0.81 | 0.59 |
| EP (J/min) | 0.77 (0.72-0.82) | <0.001 | >14.6 | 0.75 | 0.66 |

AUC, the area under receiver operating characteristic; Pplat, plateau pressure; ΔP, driving pressure; Cst, static lung compliance; MP, mechanical power; EP, elastic power.

**Table S6** Factors associated with moderate-severe ARDS by of binary logistic regression analysis.

| **Variables** | **Univariable analysis** | | **Multivariable model** | |
| --- | --- | --- | --- | --- |
|  | **HR (95% CI)** | ***p* value** | **HR (95% CI)** | ***p* value** |
| EP (J/min) | 1.21 (1.15-1.28) | <0.001 | 1.20 (1.14-1.28) | <0.001 |
| [4ΔP+RR] | 1.02 (1.01-1.03) | 0.004 | 1.02 (1.00-1.03) | 0.015 |

ΔP: Driving pressure, EP: elastic power, RR: respiratory rate

Multivariable model was adjusted for age, BMI and SOFA score.


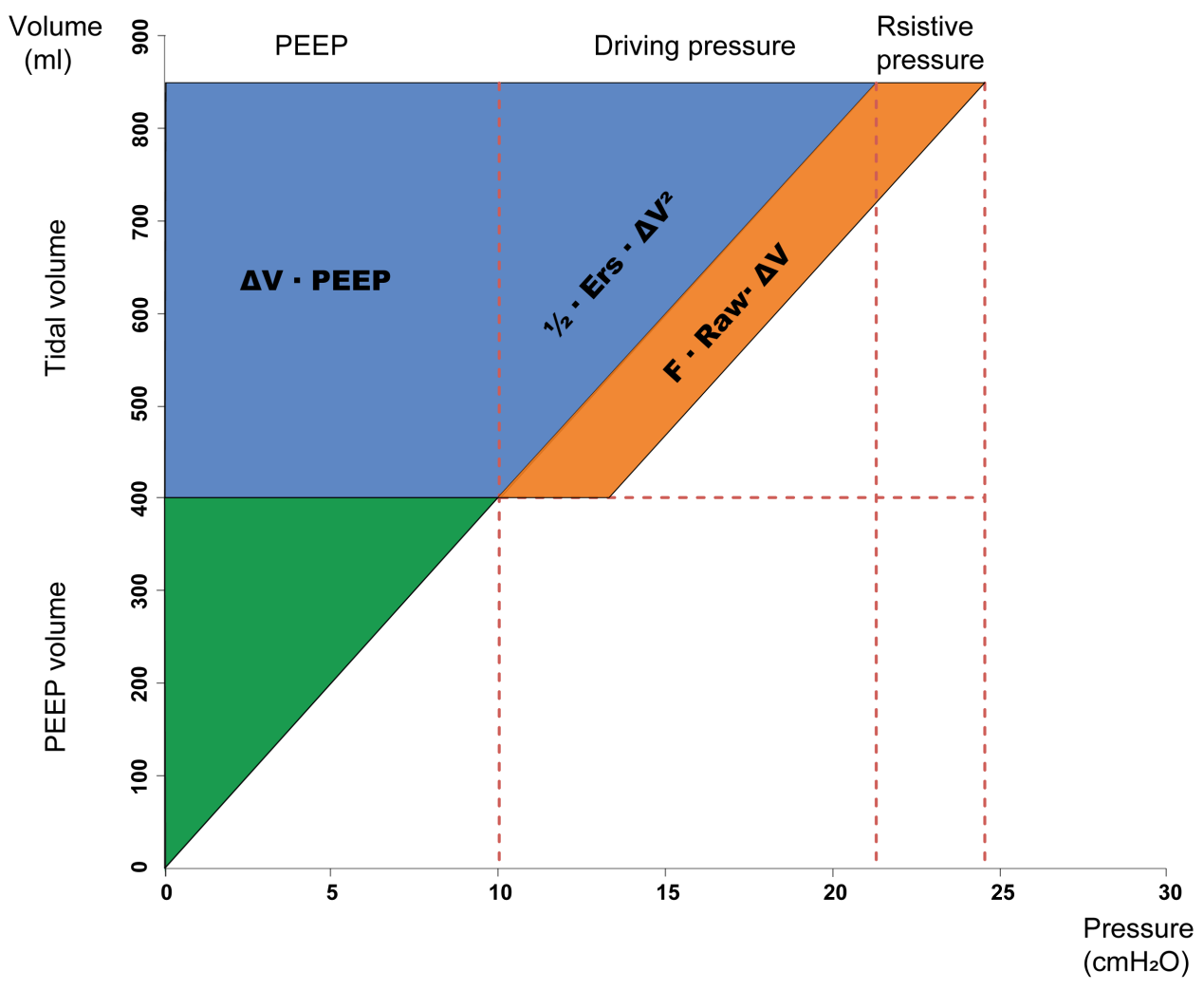
**Fig. S1 Graphical representation of the equation of power[11].**

Graphical representation of the equation of power. The upper left rectangle represents the baseline stretch of the fibers, the energy level to be overcome at each tidal volume delivery (Component 1. Elastic–static power related to PEEP: elastic static power (J/min) = 0.098×VT×RR×PEEP). The blue triangle represents the energy needed to win the elasticity of the respiratory system(Component 2. Elastic–dynamic power, related to DP: elastic dynamic power (J/min) =0.098×VT×RR×½×DP). EP is the sum of Component 1 and Component 2 (elastic static power+elastic dynamic power).The orange parallelogram represents the energy needed to win the resistance to the gas flow. The lower green triangle represents the static component of PEEP (PEEP multiplied by the PEEP volume), not taking part in the equation of power, as it is delivered just once (at the first application/change of PEEP).

**
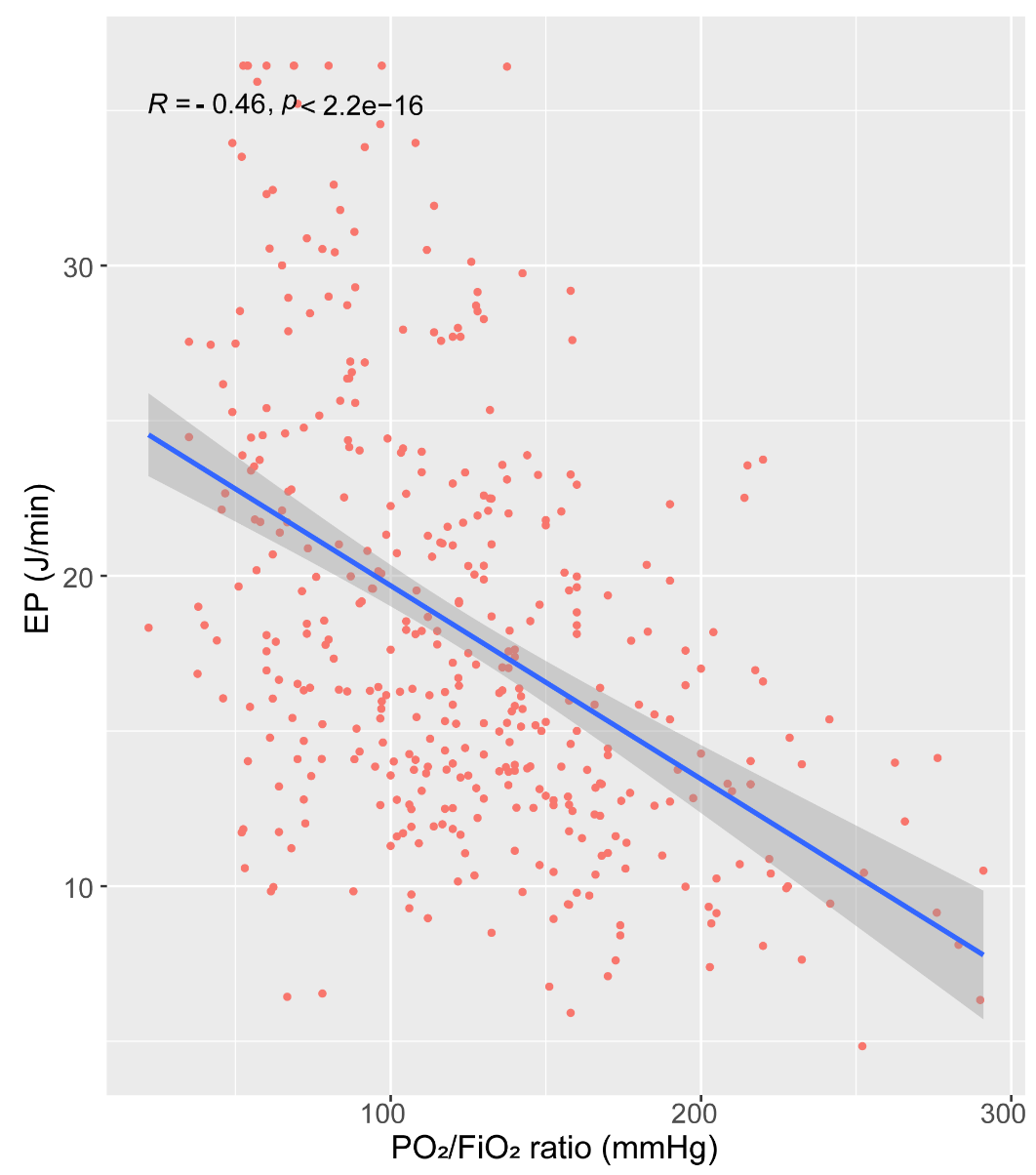
**

**Fig. S2 Correlation Between EP and PaO_2_/FiO_2_ ratio in ARDS Patients.**


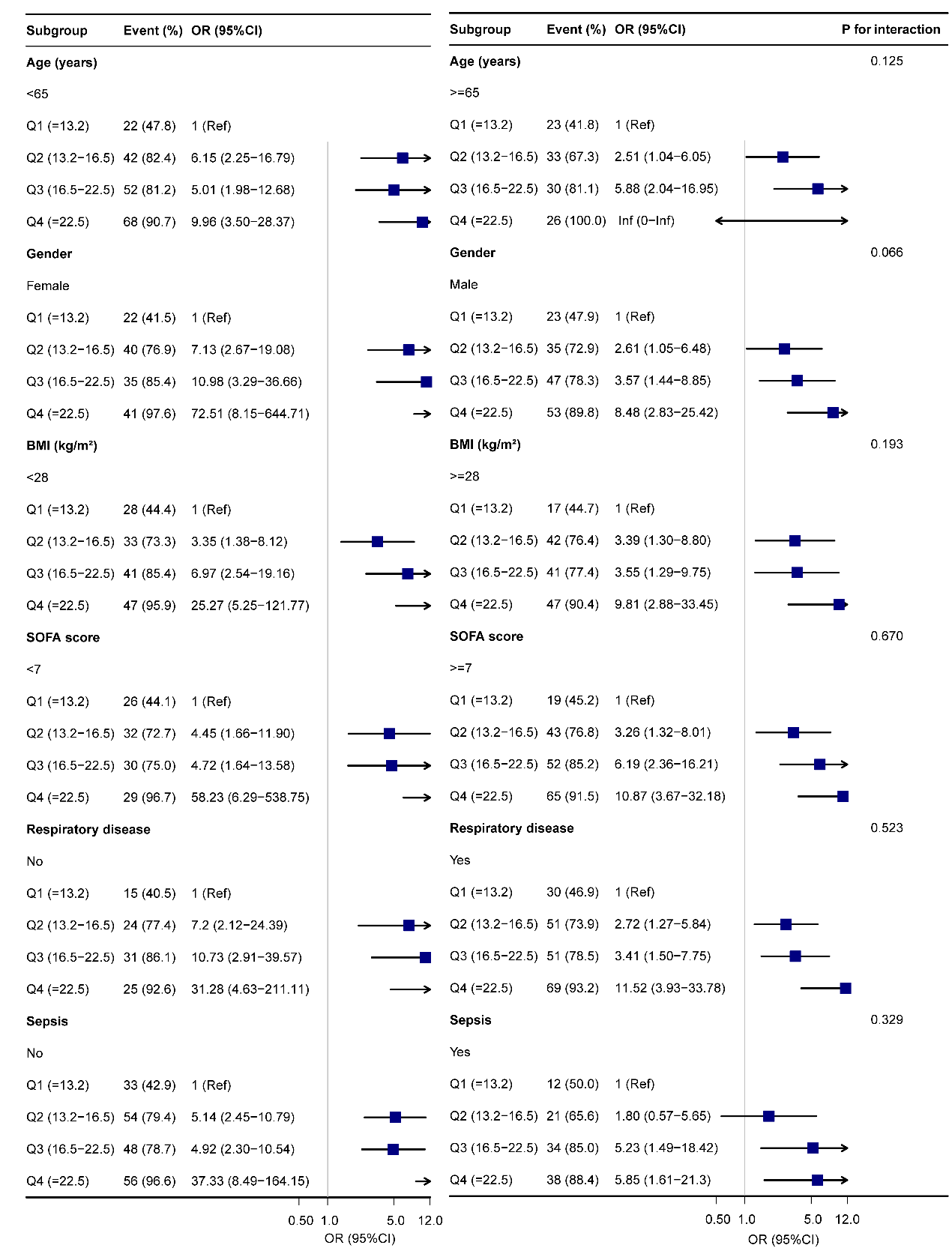


**Fig. S3 The relationship between elastic power and moderate-severe ARDS according to basic features. Each stratification was adjusted for age, BMI, SOFA score, MBP and PCO_2_ except the stratification factor itself. Squares indicate odds ratios (ORs), with horizontal lines indicating 95%CIs.**


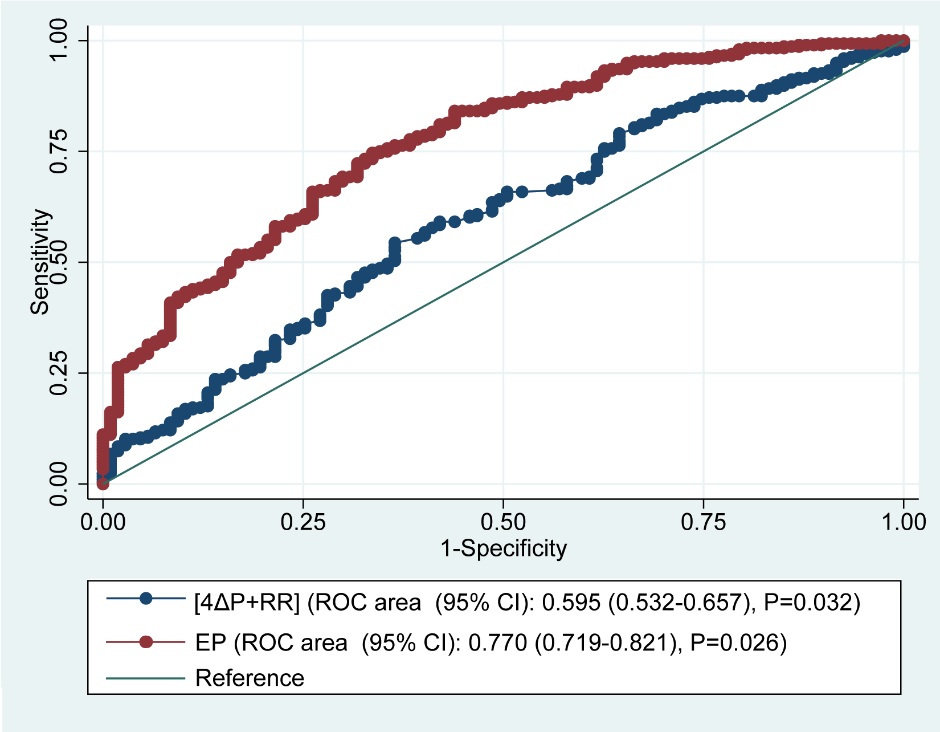


**Fig. S4 Comparision of Receiver operating characteristic curves for [4ΔP+RR] and EP.**

**ΔP: Driving pressure, RR: respiratory rate, EP: elastic power**
